# Does each element of the sepsis resuscitation bundle equally improve patient outcome?

**DOI:** 10.1186/cc9706

**Published:** 2011-03-11

**Authors:** B Afessa, MT Keegan, GE Schramm, O Gajic

**Affiliations:** 1 Mayo Clinic, Rochester, MN, USA

## Introduction

The Institute for Healthcare Improvement advocates the use of bundles to implement the sepsis guidelines. There are limited data addressing which elements improve survival [1]. We analyzed the data from a previous study to determine the independent impact of each element on patient outcome. We hypothesized that not all elements of the bundle have equal impact on outcome.

## Methods

The seven elements of the sepsis resuscitation bundle include lactate measurement, blood culture before antibiotic, timely antibiotic, adequate fluid resuscitation, appropriate vasopressor use, appropriate red blood cell (RBC) transfusion, and appropriate inotrope use. Baseline variables and the elements of the resuscitation bundle associated with mortality by univariate analyses at *P *< 0.1 were included the propensity score. The univariate associations between the baseline variables and mortality were obtained from our previous study. The propensity scores were estimated using multiple logistic regression analysis.

## Results

The study included 962 patients. Lactate measurement, timely blood culture and antibiotic administration, appropriate fluid resuscitation, and appropriate inotrope use were associated with increased mortality at *P *< 0.1 using univariate analyses. Using the propensity score of each bundle element for adjustment, compliance with lactate measurement and inotrope administration were independently associated with decreased risk of death (Table 1). Timely antibiotic administration had a trend toward risk reduction, the *P *value did not reach statistical significance. Obtaining blood culture before antibiotic administration, vasopressor administration, and RBC transfusion were not associated with decreased risk of death.

**Table 1:** 

Bundle element	OR (95% CI)	*P *value
Lactate	0.581	0.022
Antibiotic	0.706	0.085
Inotrope	0.678	0.033

## Conclusions

Using the propensity score to adjust for compliance with each bundle element, lactate measurement and inotrope administration were independently associated with reduced risk of death.

## References

[B1] Ferrer R JAMA 2008 299 2294 2303 10.1001/jama.299.19.2294 18492971

